# Safety and Efficacy of Uninterrupted Oral Anticoagulation in Patients Undergoing Catheter Ablation for Atrial Fibrillation with Different Techniques

**DOI:** 10.3390/jcm12206533

**Published:** 2023-10-15

**Authors:** Marco Schiavone, Alessio Gasperetti, Annalisa Filtz, Gaia Vantaggiato, Cecilia Gobbi, Claudio Tondo, Giovanni Battista Forleo

**Affiliations:** 1 Cardiology Unit, Luigi Sacco University Hospital, 20131 Milan, Italy; annalisa.filtz@unimi.it (A.F.); gaiavantaggiato@hotmail.it (G.V.); forleo@me.com (G.B.F.); 2 Department of Clinical Electrophysiology and Cardiac Pacing, Centro Cardiologico Monzino, IRCCS, 20138 Milan, Italy; claudio.tondo@cardiologicomonzino.it; 3 Department of System Medicine, University of Rome Tor Vergata, 00133 Rome, Italy; 4 Department of Cardiology, Johns Hopkins University, Baltimore, MD 21218, USA; 5 Department of Cardio-Thoracic-Vascular Diseases, Foundation IRCCS Ca’ Granda Ospedale Maggiore Policlinico, 20154 Milan, Italy; cecilia_gobbi@hotmail.it; 6 Department of Biomedical, Surgical and Dental Sciences, University of Milan, 20122 Milan, Italy

**Keywords:** atrial fibrillation, catheter ablation, oral anticoagulation, uninterrupted, pulmonary vein isolation

## Abstract

Background. The safety and efficacy of an uninterrupted direct anticoagulation (DOAC) strategy during catheter ablation (CA) for atrial fibrillation (AF) has not been fully investigated with different ablation techniques. Methods. We evaluated consecutive AF patients undergoing catheter ablation with three different techniques. All patients were managed with an uninterrupted DOAC strategy. The primary endpoint was the rate of periprocedural thromboembolic and bleeding events. The secondary endpoints of the study were the rate of MACE and bleeding events at one-year follow-up. Results. In total, 162 patients were enrolled. Overall, 53 were female and the median age was 60 [55.5–69.5] years. The median CHA_2_DS_2_-VASc and HAS-BLED scores were 2 [1–4] and 2 [1–2], respectively. In total, 16 patients had a past stroke or TIA while 11 had a predisposition or a history of bleeding. The CA procedure was performed with different techniques: RF 43%, cryoballoon 37%, or laser–balloon 20%. Overall, 35.8% were on rivaroxaban, 20.4% were on edoxaban, 6.8% were on apixaban, and 3.7% were on dabigatran. All other patients were all naïve to DOACs; the first anticoagulant dose was given before the ablation procedure. As for periprocedural complications, we found three groin hematomas not requiring interventions, one ischemic stroke, and one systemic air embolism (the last two likely due to several catheter changes through the transeptal sheath). Five patients reached the secondary endpoints: one patient for a myocardial infarction while four patients experienced minor bleeding during 1-year follow-up. Conclusions. Our results corroborate the safety and the efficacy of uninterrupted DOAC strategy in patients undergoing CA for AF, regardless of the ablation technique.

## 1. Introduction

Catheter ablation (CA) for atrial fibrillation (AF) is an effective and safe treatment option in the rhythm control strategy of this arrhythmia. Approximately 4–14% of patients undergoing AF catheter ablation may experience periprocedural complications. Among those, 2–3% are potentially life-threatening depending on the technique used, management characteristics, and operators’ expertise [1,2]. Nevertheless, the overall periprocedural mortality rate is about <0.1% [3]. This procedure-related complication rate has significantly decreased during the most recent 5-year period (3.77% vs. the previous 5-year period of 5.31%), as shown in a recent systematic review and pooled analysis from Benali et al. This analysis, including 15,701 patients, showed that overall and severe procedure-related complication rates were 4.51% and 2.44%, respectively, with vascular complications being the most frequent type of complication (1.31%). Most major frequent intra- and perioperative complications are either hemorrhagic or thromboembolic, such as vascular access bleeding, pericardial effusion, cardiac tamponade (in 1–3% of cases), TIA (transient ischemic attack), or stroke (1%). In order to minimize the procedure-related thromboembolic risks, it is necessary to maintain the patient on anticoagulant therapy before, during, and after the CA [4,5,6,7,8,9]. During the procedure, the required anticoagulation state is achieved by the administration of unfractionated heparin, ideally prior to or immediately following transseptal puncture, and adjusted to maintain a target activated clotting time (ACT) of 300 s or more. Regarding the management of OAC therapy, as long as indicated by the European Society of Cardiology’s Guidelines (ESC), oral anticoagulation therapy should be administered without interruption (Level Ia recommendation) in patients undergoing CA for AF [10]. This strategy has been shown to be non-inferior to vitamin K antagonists (VKAs) in stroke prevention also with a significant reduction in bleeding events (risk of 0.9% with DOACs vs. 2% with VKAs) [11,12,13,14,15,16]. 

However, in real-world practice, this strategy still worries some cardiac electrophysiologists who use another therapeutic option called the “minimally interrupted administration” in which the dose of the direct oral anticoagulation therapy (DOACs) preceding the CA procedure is suspended, in order to avoid higher bleeding risks [8]. Despite ESC recommendations, this strategy is often used in real-world practice due to bleeding concerns, especially in patients with high bleeding risks or whenever an extensive ablation lesion set is anticipated. Indeed, the evidence behind the use of an uninterrupted anticoagulation protocol is mostly derived from randomized trials on VKAs, while there are only a few focused reports on DOACs mostly analyzing radiofrequency procedures. Therefore, our study aims to evaluate the safety and the efficacy of uninterrupted oral anticoagulation using direct oral anticoagulants (DOAC) in a real-world setting in patients undergoing CA for AF with different techniques (radiofrequency, cryoballoon—CB, and laser balloon—LB).

## 2. Materials and Methods

### 2.1. Registry Population

We retrospectively evaluated all patients admitted to our Cardiology Unit (Luigi Sacco University Hospital) between November 2019 and April 2022. All consecutive patients meeting current guideline indications for CA for AF with no contraindications to DOAC therapy and who underwent AF ablation in our laboratory were used for the current analysis. Patients affected by moderate or severe hepatic impairment, end-stage kidney failure (GFR < 15 mL/min), pregnancy, or breastfeeding were excluded. This manuscript was drafted in accordance with the tenets of the Helsinki Declaration and conducted according to the local ethics committee regulations. Data supporting this study are available upon reasonable request to the corresponding author. 

### 2.2. Data Collection, Study Cohort, and Outcomes Definition

The demographics, patient medical histories, lab analyses, and peri-procedural data were collected through the digital archive of the hospital. For each patient CHA_2_DS_2_-VASc and HAS-BLED scores were calculated. All patients underwent a transthoracic echocardiogram prior to the procedure to evaluate the ejection fraction, the left atrial (LA) dimension, and to exclude the presence of pericardial effusion. Moreover, patients presenting with AF on the day of the procedure underwent a transesophageal echocardiographic evaluation to exclude left atrial appendage thrombosis. Each procedure was performed either under general anesthesia or in deep sedation, as appropriate. Regarding the management of the anticoagulation therapy, according to the current ESC guidelines, all patients underwent an uninterrupted anticoagulation protocol, as per our center policy. For each patient, the EP team chose the most appropriate technique for CA (radiofrequency, CB, or LB). The procedure was performed by the catheterization of both femoral veins which allowed the introduction of different catheters (depending on the technique), namely a decapolar catheter, and an intracardiac ultrasound (ICE, intracardiac echocardiography). 

The transseptal puncture was guided by a combination of ICE and radioscopic techniques to achieve left atrial access. During this specific phase of the ablation procedure, refracted boluses of unfractionated heparin were administered to reach an ACT (activated clotting time) > 300 s. Boluses were eventually repeated every 15 min according to the ACT value. At the end of the procedure, when the ACT reached a value lower than 150 s either spontaneously or after the administration of protamine sulfate, we removed the sheaths applying manual compression or a suture-of-eight. The presence of pericardial effusion was checked either with ICE or with transthoracic echocardiography. At three months after the procedure, all the patients were planned to a follow-up visit in the outpatient clinic.

The primary endpoint was the rate of periprocedural thromboembolic and bleeding events. The secondary endpoints of the study were (1) the detection of the combined incidence of major adverse cardiovascular events (MACE-3 or three-point major adverse cardiovascular events) at one year of follow-up and (2) the combined rate of major and minor bleeding events at one year follow-up. As indicated by the International Society on Thrombosis and Hemostasis guidelines [17], we considered “severe bleeding” as symptomatic bleeding leading to death and/or bleeding in a critical area or organ (e.g., intracranial, intraspinal, intraocular, retroperitoneal, intra-articular or pericardial, or intramuscular with compartment syndrome) and/or a bleeding associated with a drop of the hemoglobin (Hb) level greater than 2 g/dL or a severe reduction of the Hb value, requiring the transfusion of two or more units. All the other bleeding events not fulfilling the aforementioned criteria were classified as non-severe bleeding. In terms of thromboembolic events, we accounted for the presence of stroke following the definitions of the Society of Neurology and for cardiovascular deaths, defined as “death caused by myocardial infarction, sudden cardiac death, heart failure, stroke, cardiovascular procedure, hemorrhage and other cardiovascular causes” [18,19].

### 2.3. Statistical Analysis

The reported variables were tested for normality with the Shapiro–Wilk test. Continuous variables are expressed as the mean ± standard deviation (SD) or as the median [IQR] (1st–3rd quartile) based on normal or non-normal distribution, respectively. Discrete variables are expressed as a number (%). Data analysis was performed using STATA 14.0 (StataCorp LLC, College Station, TX, USA).

## 3. Results

### 3.1. Baseline Characteristic of the Study Cohort

A total of 173 patients were initially enrolled in the current study; 11 subjects were excluded for not attending the follow-up visit within three months of the procedure. Baseline characteristics of the study cohort are summarized in Table 1. Among the 162 patients, 53 were female, with a median age at the time of the procedure of 60 [55.5–69.5] years. The more frequent comorbidity was hypertension (n = 114) followed by chronic kidney disease (n = 29), chronic heart failure (n = 16), and diabetes (n = 16). Overall, 65 patients were affected by structural heart disease, better defined as follows: ischemic cardiopathy (n = 25), valvular heart disease (n = 14), hypertensive cardiomyopathy (n = 13), non-ischemic dilated cardiomyopathy (n = 8), and high-rate AF-induced tachycardiomyopathy (n = 5). The CHA_2_DS_2_-VASc and HAS-BLED scores presented a median value of 2 [1–4] and 2 [1–2], respectively. The maximum CHA_2_DS_2_-VASc score was eight while the maximum HAS-BLED score recorded was five. A history of past stroke or TIA was reported in 16 patients while 11 subjects had a predisposition or a previous history of bleeding. A total of 79 patients had already undergone an electrical or pharmacological cardioversion, prior to this study.

Regarding the AF baseline pattern, 119 patients had paroxysmal AF, 23 had persistent AF, and 20 patients were affected by a long-standing persistent form. A total of 108 patients of our cohort were on DOAC prior to the ablation procedure. Among the DOACs, 58 subjects were on rivaroxaban (35.8–81% with full dose), 33 on edoxaban (20.4–82% with full dose), 11 on apixaban (6.8–91% with full dose), and 6 patients on dabigatran (3.7–6.8% with full dose). The other patients were all naïve to DOACs and the first anticoagulant dose was given before the ablation procedure. A total of 79 patients were not on antiarrhythmic therapy while 37 patients were on class IC antiarrhythmic drugs (flecainide or propafenone) and 45 on amiodarone; only one patient was on sotalol. A comprehensive description of the baseline therapy of our study cohort is summarized in Table 2.

### 3.2. Transcatheter Ablation Procedure

The CA procedures were performed with different techniques: radiofrequency ablation (RF) (n = 70 cases; 43%), CB ablation (n = 60 cases; 37%), or LB ablation (n = 32 cases; 20%). All cases underwent pulmonary vein isolation (PVI); other additional ablation lesions were performed in 32 cases (additional posterior wall [PW] ablation in 16 cases and PW + additional lesions such as roof lines and/or mitral annulus in the other 16 cases). Femoral venous accesses were always obtained with ultrasound guidance (number of median accesses = 3 and number of maximum accesses = 4). A total of 156 accesses were closed by manual compression while in 6 cases a “point-of-eight” suture was performed. All transeptal punctures were achieved by the ICE guidance. The median dose of heparin administered during the procedures was 8000 IU while protamine was administered in 40 procedures (64.5%). 

When considering periprocedural complications, we collected three groin hematomas not requiring further interventions, one case of ischemic stroke, and one case of systemic embolism. The ischemic stroke occurred during a procedure characterized by several catheter introductions and removals through the transeptal sheath (specifically removing and reintroducing the LB and a multipolar mapping catheter through the LB sheath). The systemic air embolism was diagnosed due to ST elevation requiring an urgent coronary angiography. Both events were solved without clinical sequelae. The overall periprocedural complications are reported in Table 3.

### 3.3. Follow-Up

All patients underwent a follow-up evaluation at 3, 6, and 12 months after the procedure, as per our center policy. During the programmed follow-up, a total of 103, 95, and 84 patients were still on anticoagulation therapy at the different timeframes. Data collected throughout the follow-up are summarized in Table 4. A total of five patients reached the secondary endpoints: one patient experienced a myocardial infarction while four patients experienced minor bleeding (n = 2 respiratory tract bleeding and n = 2 experiencing macroscopic hematuria) during the 1-year follow-up.

## 4. Discussion

This is a monocentric and retrospective study evaluating the efficacy and the safety of the uninterrupted anticoagulation regimen during catheter ablation for AF [20] in a real-world setting using different ablation techniques. Our results corroborate the safety and efficacy of the uninterrupted DOAC strategy in patients undergoing CA for AF, regardless of the technique used [21,22]. Thus, this is the first report analyzing the safety of uninterrupted anticoagulation in a mixed cohort of radiofrequency, CB, and LB patients and one of the first enrolling patients who were naïve to OACs before ablation as well. We observed an incidence of 1.2% of major periprocedural complications represented by embolic events (one periprocedural ischemic stroke and one systemic air embolism). These data are consistent overall with other reports in the literature [4,5,6,7,8,9,10].

### 4.1. Periprocedural Embolic Events

The increased risk of thrombus formation during CA has been widely documented even though the exact mechanism has not been completely understood yet [23]. Both physical stimulation and high- or low-temperature changes, performed during CA with LB or CB, respectively, may play a role in changing the blood coagulation status more than with RF ablation [24,25,26]. Indeed, as referred to by Okishage et al., the blood coagulation activity is also enhanced during the extremely low blood temperatures reached by CB ablation [27]. These data are congruous with the results from a sub analysis of the Kyurable study, documenting a greater increase in the coagulation biomarkers in the CB than the RF group [28]. However, no significant differences in the incidence of thrombotic or bleeding events were reported, supporting the safety use of DOAC during CA with both RF or CB [28,29]. No data on LB ablation have been reported so far; our study did not find any relevant difference among groups regarding ischemic periprocedural events, corroborating the lack of differences among techniques and thereby supporting the idea that the underlying mechanism has to be found elsewhere.

In our study, only one patient (0.6%) experienced a periprocedural thromboembolic complication. These data are consistent with the incidence of stroke secondary to ablation reported by the current literature (between 0.4% and 2.1%) [30], reaching the lower limits of the aforementioned range. The Apixaban During Atrial Fibrillation Catheter Ablation (AXAFA–AFNET 5) randomized clinical trial (RCT), comparing uninterrupted apixaban vs. warfarin, found two strokes in 633 patients (0.3%) [14] compared to one stroke in 248 patients in the VENTURE-AF (Uninterrupted rivaroxaban vs. uninterrupted vitamin K antagonists for catheter ablation in non-valvular atrial fibrillation) trial (0.4%) [15], one TIA in 635 patients in the RE-CIRCUIT (Uninterrupted Dabigatran versus Warfarin for Ablation in Atrial Fibrillation) trial (0.2%) [16], and one stroke in 411 patients in the ELIMINATE AF (Uninterrupted Dabigatran versus Warfarin for Ablation in Atrial Fibrillation) trial (0.2%) [13]. In all these studies, the incidence of thromboembolic events was lower in the DOAC group than in the warfarin group. The other two Japanese RCTs showed similar findings under uninterrupted DOACs compared with VKAs [28,31]. In a meta-analysis of 12 studies, uninterrupted anticoagulation using DOACs vs. VKAs for AF catheter ablation was associated with low rates of stroke/TIA (NOACs, 0.08%; VKA, 0.16%) and similar rates of silent cerebral embolic events (8.0% vs. 9.6%) [11]. Moreover, the recent CABANA (Catheter ABlation vs. ANtiarrhythmic Drug Therapy for Atrial Fibrillation) trial has shown that adverse procedural complications associated with catheter ablation were less likely to occur when the procedure is performed by experienced operators [32]. Indeed, the centers that reported the lowest incidence of stroke/TIA are the ones with the highest volume procedures, the most experienced operators, and the ones using the uninterrupted anticoagulation protocol, as in our center. This unique periprocedural thromboembolic complication of our registry occurred during an LB CA and electro-anatomical mapping. The procedure was characterized by multiple catheter changes through the transseptal introducer, due to several balloon changes as well as pre- and post-ablation mapping. This patient had a low thromboembolic risk (CHA_2_DS_2_-VASc = 0) and no past medical history of stroke or TIA. Therefore, it could be assumed that this specific adverse event was related to the multiple catheter changes through the same transseptal introducer although the catheters were correctly flushed as per our lab’s strict protocol. According to this panel of authors, this mechanism, more than the previously discussed potential alteration of the blood temperature, has to be considered the most important determinant of periprocedural thromboembolic complications during catheter ablation for AF.

In addition to this single case of peri-procedural stroke, we reported one case (0.6%) of systemic air embolism. This procedure, performed with the RF technique, was complicated by an ST elevation and diagnosed with procedural ECG continuous monitoring. An urgent coronary angiography was performed documenting the presence of an air bubble in the circumflex artery which spontaneously resolved. The possible mechanisms of the air embolisms during ablation were considered to be related to gaseous emboli caused by the introduction of air through the sheath during the prolonged manipulation and the multiple substitution of the catheters or due to residual air attached to the instruments due to insufficient removal of air bubbles more than to a thromboembolism provoked by the activation of the coagulation cascade [33]. 

Indeed, the introduction and removal of a catheter through the transseptal introducer increases the risk of silent cerebral infarction (SCI) compared to performing a second transseptal puncture [34]. Thus, this technique is related to the potential introduction of thrombotic material or air bubbles into the introducer catheter itself and into the left atrium. On the other hand, performing a second transseptal puncture increases the risks related to the puncture itself which are minimized using intracardiac ultrasound guidance, as we have systematically performed in all procedures. 

One of the greatest benefits of an uninterrupted OAC strategy during catheter ablation for AF is the lower incidence of silent ischemic events compared with interrupted anticoagulation. However, to date, the clinical relevance of silent cerebral events remains unclear since some works suggest that silent cerebral events might be associated with cognitive impairment occurring after an AF ablation procedure; but other studies have failed to demonstrate this correlation [35]. If Nakamura et al. showed that additional RF delivery within the left atrium in patients undergoing non-PV triggers is an independent risk factor for cerebral ischemic events [36], it seems that the uninterrupted OAC strategy may lead to fewer silent cerebral events [37]. Although, in our study, we did not systematically address this issue with brain imaging; no overt cognitive impairment was detected at follow-up, potentially corroborating the benefit of this strategy.

### 4.2. Periprocedural Bleeding Events

In our study, we recorded a low overall incidence of total bleeding events (0% for severe events and 2.4% for non-severe events), with no patient experiencing major bleeding events during the procedure or during hospitalization. Apart from 3 (1.6%) mild groin hematomas, there were no major complications related to the femoral access. The current guidelines reported a vascular complication rate of 2–4%; [10] our better outcome may be due to the systematic application of an ultrasound-guided approach to venous femoral accesses. Indeed, this technique increases the safety of catheterization compared to the approach based on anatomical landmarks [38]. Thus, as demonstrated by several trials and meta-analyses, the use of real-time 2D ultrasound guidance for femoral vein cannulation decreases access-related bleeding rates and life-threatening vascular complications [39,40,41,42].

Moreover, no episodes of pericardial effusion or cardiac tamponade were reported. Considering the reported rate in the literature (1%), ref. [10] this relatively low rate could be related to the sample size of our study as well as to the systematic application of the intracardiac eco-guided approach for the transseptal puncture, differently from other RCT [14,16].

One patient (0.8%) experienced an in-hospital urinary tract bleeding event due to macroscopic hematuria during bladder catheterization. This event was managed with a temporaneous suspension of OAC therapy that was then resumed soon after the bleeding was solved. Our data are consistent with the literature, reporting an incidence of non-severe periprocedural bleeding of approximately 4% and a better safety profile of uninterrupted DOACs compared to uninterrupted VKAs [10,30,43]. Indeed, non-inferiority studies concerning minor bleeding reported an OR of 0.95 in cohorts undergoing uninterrupted anticoagulation with DOAC [8]. In the Uninterrupted Dabigatran versus Warfarin for Ablation in Atrial Fibrillation (RE-CIRCUIT) trial comparing periprocedural DOAC vs. warfarin, the incidence of major bleeding events during and up to eight weeks after ablation was significantly lower with dabigatran (1.6%) than with warfarin (6.9%) [16]. Other trials comparing uninterrupted periprocedural DOACs vs. VKAs documented a significant reduction in major bleeding with direct anticoagulation compared with uninterrupted VKAs in patients undergoing AF [13,14,15,16]. Furthermore, heparin bridging increases the risk of bleeding and should be avoided.

### 4.3. Follow-Up Events

During the follow-up, a total of three cases of minor bleeding were reported. One of them was an episode of macroscopic hematuria in a patient on OAC and dual antiplatelet therapy; therefore, OAC therapy was briefly stopped and then restarted after the event. This triple therapy is associated with a three-fold increase in the absolute risk of serious bleeding events compared to single anticoagulant or antiplatelet therapy [17]. Moreover, since the patient also had a medium/high bleeding risk (HAS-BLED = 3) it is reasonable to assume that the combination of these different factors led to the minor bleeding event. In all other cases, whenever OAC therapy was stopped, this was left at physician discretion. After three months of anticoagulation (blanking period), if no AF recurrences were detected at follow-up, all OACs were stopped whenever the CHA_2_DS_2_-VASc score was zero (male sex) or one (female sex). Generally, whenever no AF recurrences were detected, OACs were stopped whenever the CHA_2_DS_2_-VASc Score was one (male sex) or two (female sex), always according to European guidelines. However, this was always left to physician discretion during follow-up. OAC dosage was reduced if renal function worsening was detected. With this management, no embolic events were recorded during the follow-up period.

### 4.4. Other Events

As previously mentioned, the centers with the highest volume of CA procedures and with experienced operators have fewer adverse events related to this invasive technique [10]. The rate of complication reported in our study is concordant with the data presented in the literature (complication rate 5%) [10,44]. As for other events, we documented three procedures complicated by phrenic nerve paralysis. Among them, two ablations were performed by the LB technique while the third was by CB ablation, with a cumulative incidence of 1.8% (1.2% for LB and 0.6% for CB, respectively), lower than the one reported in the literature (3% in the CB ablation technique). However, comparing our results to the one presented in the Get With The Guidelines-Atrial Fibrillation (GWTG-AFIB) registry, we recorded a higher incidence of phrenic nerve injury. This could be explained by the greater use of balloon catheters compared to the linear RF catheters experienced in our center compared to the GWTG-AFIB registry (56.8% vs. 23%, respectively) [44].

### 4.5. Limitations

Due to the overall low number of patients who met the study endpoints, it is arduous to correlate the results to patients’ demographic characteristics, their comorbidities, and drug therapy. For the same reasons, a survival analysis for the primary endpoints could not be performed, limiting the ultimate statistical power of this analysis. Moreover, the value of this study may be hampered by the inability to perform proper statistical comparisons between the different AF ablation techniques, in terms of periprocedural ischemic and bleeding complications, due to the low overall number of events. Furthermore, retrospective data collection may lead to incomplete or inaccurate information (although these authors exclude a significant underestimation of major periprocedural complications) and the exclusion of 11 patients who did not attend follow-up could bias the results regarding the secondary outcomes during follow-up. Lastly, since a post-procedural brain MRI was not systematically performed, it was not possible to evaluate the rate of silent cerebral infarctions and the benefit of the uninterrupted OAC strategy in this regard.

## 5. Conclusions

An uninterrupted direct oral anticoagulation strategy in patients undergoing catheter ablation for AF is effective in minimizing the risk of peri-procedural stroke or TIA while not increasing the risks of bleeding complications. Our study confirmed that uninterrupted anticoagulation is a safe and effective protocol both in radiofrequency and in balloon techniques even in real-world practice (either with the CB or with the LB), especially when procedures are performed by an expert team. Further research and clinical trials are nevertheless needed to confirm the safety and efficacy of uninterrupted OAC therapy during catheter ablation for AF.

## Figures and Tables

**Table 1:** Characteristics of the study population (n = 162).

Age, median [IQR]	60.0 [55.5–69.5]
Female sex, n (%)	53 (32.7)
Body weight, kg	79.5 ± 15.7
Height, cm	171.1 ± 9.4
BMI, median [IQR]	26.1 [24.2–29.4]
Type of Atrial Fibrillation Paroxysmal, n (%) Persistent, n (%) Long-standing persistent, n (%)	119 (73.5) 23 (14.2) 20 (12.3)
Cardiopathy Ischemic n (%) Valvular, n (%) Hypertensive, n (%) Dilated non ischemic, n (%) Tachycardiomyopathy, n (%)	25 (15.4) 14 (8.6) 13 (8.0) 8 (4.9) 5 (3.1)
Chronic Heart Failure, n (%)	22 (13.5)
LVEF (%)	62 [59–67]
Hypertension, n (%)	114 (70.4)
Diabetes, n (%)	16 (9.9)
Chronic Kidney Disease, n (%)	29 (17.9)
Creatinine mg/dL, median [IQR]	0.94 [0.81–1.10]
Liver Failure, n (%)	2 (1.2)
COPD, n (%)	5 (3.1)
History of stroke or TIA, n (%)	16 (9.9)
CHA_2_DS_2_-VASc, median [IQR]	2 [1–4]
History or risk factors for bleeding, n (%)	11 (6.8)
Concomitant antiplatelet therapy, n (%)	26 (16.0)
HAS-BLED, median [IQR]	2 [1–2]
Previous electric or pharmacological cardioversion, n (%)	79 (48.8)

BMI: body mass index, COPD: chronic obstructive pulmonary disease, AF: atrial fibrillation, LVEF: left ventricular ejection fraction, TIA: transient ischemic attack.

**Table 2:** Baseline drug therapy.

*Anticoagulation therapy* None, n (%) Rivaroxaban, n (%) *Full-dose* *Reduced dose* Edoxaban, n (%) *Full-dose* *Reduced dose* Apixaban, n (%) *Full-dose* *Reduced dose* Dabigatran, n (%) *Full-dose* *Reduced dose*	54 (33.3) 58 (35.8) 47 (81) 11 (19) 33 (20.4) 27 (82) 6 (18) 11 (6.8) 10 (91) 1 (9) 6 (3.7) 3 (50) 3 (50)
*Antiarrhythmic therapy* None, n (%) Amiodaron, n (%) AAD class IC, n (%) Sotalol, n (%)	79 (48.8) 45 (27.8) 37 (22.8) 1 (0.6)
*Other drugs* β-blockers, n (%) ACE inhibitors and ARB, n (%) Calcium channel blockers, n (%) SAPT, n (%) DAPT, n (%)	97 (59.9) 88 (54.3) 30 (18.5) 25 (15.4) 1 (0.6)

**Table 3:** Procedural data and periprocedural complications.

Type of ablation Radiofrequency, n (%) Cryoballoon, n (%) Laser balloon, n (%)	70 (43.2) 60 (37.0) 32 (19.8)
N. of transseptal puncture 1, n (%) 2, n (%)	122 (75.3) 40 (24.7)
Ablation lesion set PVI alone, n (%) PVI + PW, n (%) PVI + PW + other lines, n (%)	130 (80.2) 16 (9.9) 16 (9.9)
Anesthesia Deep sedation, n (%) general anesthesia with intubation, n (%)	147 (90.7) 15 (9.3)
Number of femoral venous access, median [IQR]	3 [3–4]
Eco-guided femoral access, n (%)	162 (100)
Femoral access closure Manual compression, n (%) Figure-of-eight suture, n (%) Vascular closure systems, n (%)	130 (96.3) 6 (3.7) 0 (0)
Femoral access for blood pressure monitoring, n (%)	11 (6.8)
Eco-guided transseptal puncture, n (%)	162 (100)
Intraprocedural heparin dose IU, median [IQR]	8000 [6000–9000]
Protamine at the end of the procedure, n (%)	40 (23.5)
Procedural time, median [IQR]	105 [87–140]
Fluoroscopy time, median [IQR]	21 [14–29]
Periprocedural complications Groin hematoma, no intervention needed, n (%) Groin hematoma, intervention needed, n (%) Arterio-venous femoral fistula, n (%) Periprocedural stroke, n (%) Systemic embolism, n (%) Pleural effusion, n (%) Cardiac tamponade, n (%) Urinary tract bleeding, n (%) Phrenic nerve palsy, n (%)	3 (1.8) 0 (0) 0 (0) 1 (0.6) 1 (0.6) 0 (0) 0 (0) 1 (0.6) 3 (1.8)

PVI: pulmonary vein isolation, PW: posterior wall.

**Table 4:** Time-sensitive events.

Patients on OAC during the *follow-up* At 3 months, n (%) At 6 months, n (%) At 12 months, n (%)	103 (63.6) 95 (58.6) 84 (51.9)
Patients who had to reduce OAC dose during follow-up Rivaroxaban, n (%) Edoxaban, n (%) Apixaban, n (%) Dabigatran, n (%)	3 (5) 2 (6) 0 (0) 0 (0)
MACE-3 during il follow-up Ischemic stroke or TIA, n (%) Myocardial infarction, n (%) Cardiovascular death, n (%)	0 (0) 1 (0.6) 0 (0)
Bleeding events during follow-up Major bleeding (definition ISTH), n (%) Minor bleeding, n (%) *Airways, n* (%) * Urinary tract, n* (%) * Gastrointestinal, n* (%)	0 (0) 4 (2.5) *2 (1.2)* *2 (1.2)* *0 (0.6)*
Hospitalization due to cardiac event, n (%) Hospitalization due to AF/FLA, n (%)	29 (17.9) 21 (11.7)
Recurrences AF/FLA, n (%) At 3 months, n (%) At 12 months, n (%)	21 (12.9%) 11 (6.8%)

FLA: atrial flutter, ISTH: International Society on Thrombosis and Hemostasis, OAC: oral anticoagulation, MACE-3: major adverse cardiovascular events, nonfatal stroke aggregation, nonfatal myocardial infarction, and cardiovascular death.

## Data Availability

Data supporting this study are available upon reasonable request to the corresponding author.
